# Risk of viral failure after simplification therapy without using integrase inhibitors compared with maintenance of triple antiretroviral therapy: A systematic review and meta-analysis

**DOI:** 10.1016/j.bjid.2024.104463

**Published:** 2024-11-17

**Authors:** Mateus Swarovsky Helfer, Eduardo Sprinz

**Affiliations:** a Hospital de Clínicas de Porto Alegre, Departamento de Doenças Infecciosas, Porto Alegre, RS, Brazil; b Universidade Federal do Rio Grande do Sul, Programa de Pós-Graduação em Ciências Médicas, Porto Alegre, RS, Brazil; c Universidade Federal do Rio Grande do Sul, Faculdade de Medicina, Porto Alegre, RS, Brazil

**Keywords:** Hiv, Aids, Haart, Dual therapy, Simplification, Virological failure, Boosted protease inhibitor

## Abstract

**Background:**

Antiretroviral drug simplification is a strategy to reduce drug exposure and improve treatment adherence. Nowadays, dolutegravir plus lamivudine is the most preferred regimen, which might lead in the future with problems related to drug resistance or drug intolerance. This meta-analysis aimed to assess the safety of HAART simplification without integrase inhibitors.

**Methods:**

We conducted a systematic review and meta-analysis using the Embase and Medline databases to include clinical trials and observational studies published between 2008 and March 2024. The studies focused on HIV-positive individuals with suppressed viral load who either simplified their treatment to dual therapy without integrase inhibitors or continued triple therapy. The primary outcome of interest was the likelihood of viral failure within 48 weeks.

**Results:**

Ten studies were included, with a total of 1,977 patients. Boosted Protease Inhibitors (PI) were the core of antiretroviral simplification therapy. The simplification group did not show an increased risk of virological failure, with a pooled RR in 48 weeks of 1.29 (0.61‒2.73, I² = 51 %) when compared to control group. Boosted protease inhibitors were preferred combined with lamivudine, nevirapine, efavirenz, and maraviroc). Only maraviroc plus boosted PI combination was associated with a higher risk of virological failure with an RR of 4.49 (1.99‒10.11).

**Conclusion:**

Simplification therapy with boosted PI plus lamivudine or non-nucleoside transcriptase reverse inhibitors was a safe strategy and not associated with a higher risk of virological failure. This approach might be an alternative to dolutegravir-based simplification regimens if needed.

## Introduction

The achievement of sustained viral suppression with Highly Active Antiretroviral Therapy (HAART) dramatically changed the natural history of HIV infection.^1^^,^^2^ Unfortunately, in some cases this accomplishment has been associated to adverse effects, which may lead to problems related to adherence, treatment interruption,^3^ drug intolerance and the development of comorbidities.^4^^,^^5^ As a consequence, even in the early years of HAART, treatment simplification to reduce adverse events and to make a more friendly regimen has been pursued. The goal was always to reduce the pill burden, adverse effects, and the number of antiretroviral agents while still maintaining viral suppression.

Nevertheless, the pioneer's studies failed to demonstrate the efficacy of maintaining virologic suppression upon treatment simplification. However, they provided valuable insights about maintenance therapy.^6^, ^7^, ^8^ High HIV viral load at the beginning of treatment and previous use of zidovudine, for example, have shown to be predictors of failure to maintain viral suppression in simplified patients. This illustrates certain limitations of the antiretroviral agents used at that time. Problems related to their pharmacokinetics properties, potency, and low genetic barrier contributed to the failure of the strategy of drug simplification at that time. Since this approach was inferior to HAART maintenance, dual therapy was no longer tried.

The development of newer antiretrovirals in the coming years, start to overpass issues related to pharmacokinetics properties and genetic barrier limitations. Sustained viral suppression could be achieved with less pill burden, taken once or twice daily.^9^, ^10^, ^11^ The utilization of Protease Inhibitors with low dose ritonavir (PI/r) improved their pharmacokinetic profile, with the advantage of higher half-life, lower pill burden and a higher genetic barrier against HIV-resistant mutations.^10^^,^^11^ Treatment was more friendly and with greater efficacy.^9^^,^^12^ Nevertheless, some problems remained, such as drug adverse effects and the development of comorbidities not previously related to AIDS.^13^

The lasts PI in clinical use took these advancements even further, particularly Darunavir (DRV), which has greater genetic barrier, potency, and pharmacokinetic profile,^14^^,^^15^ Still, some problems regarding to drug intolerance and drug interactions remained. The development of Integrase Strand Transfer Inhibitors (INSTI), the most potent antiretroviral class so far,^16^^,^^17^ have brought additional improvements, with less drug interactions and much better tolerated in general. The introduction of second-generation INSTIs (dolutegravir and bictegravir) and their better pharmacokinetic profile and higher potency was a step even forward.^18^^,^^19^

A new window of opportunity has been open to simplify HAART because of the improvement of antiretrovirals drugs. Initially, regimens contained PI/r plus lamivudine (3TC) or Non-Nucleoside Reverse Transcriptase Inhibitors (NNRTI). These regimens were safe and maintained long-term viral suppression. Nowadays, DTG and 3TC or PI/r (DRV) are amongst the most simplified antiretroviral regimens. Nevertheless, there are growing concerns about the use of DTG worldwide. The widespread use of DTG^20^, ^21^, ^22^ as initial or rescue therapy and the growing prevalence of integrase-associated resistance mutations might increase in the coming years. Although this possibility is not common, some recent reports^23^^,^^24^ have found a prevalence of around 6 % of major integrase resistance-associated mutations. Not only resistance could be a problem, but also up to 16 % of patients may still present some drug intolerance issues associated to DTG.^25^^,^^26^ Therefore, there will be situations in which therapy simplification with DTG may not be an option, and other alternatives would be necessary. This review aims to assess the risk of treatment simplification in this scenario.

## Methods

### Search strategy

We conducted a search in March 2024 in the MEDLINE and Embase databases. The search terms used were “HIV”, “AIDS”, “antiretroviral therapy”, “HAART”, “acquired immunodeficiency syndrome”, or “highly active antiretroviral therapy”, combined with “dual”, “double therapy”, “double antiretroviral therapy”, “two-drug”, “nucleoside-sparing”, “NRTI-sparing”, “2-drug”, “2 drug”, “two drug”, “two-drug”, “simplification”, and “switch”. Articles published between 2008 and the date of the search in English were included. The filters “clinical trials”, “clinical studies”, and “cohort analysis” were applied.

### Study selection

The study should meet all the following pre-specified inclusion criteria to be eligible: (a) Clinical trials or cohort, (b) With adults living with HIV, (c) In which the intervention or exposure was dual antiretroviral therapy, (d) The comparator was triple antiretroviral therapy, (e) And the outcome was viral suppression rate or failure in 48 weeks.

**Fig. 1:**
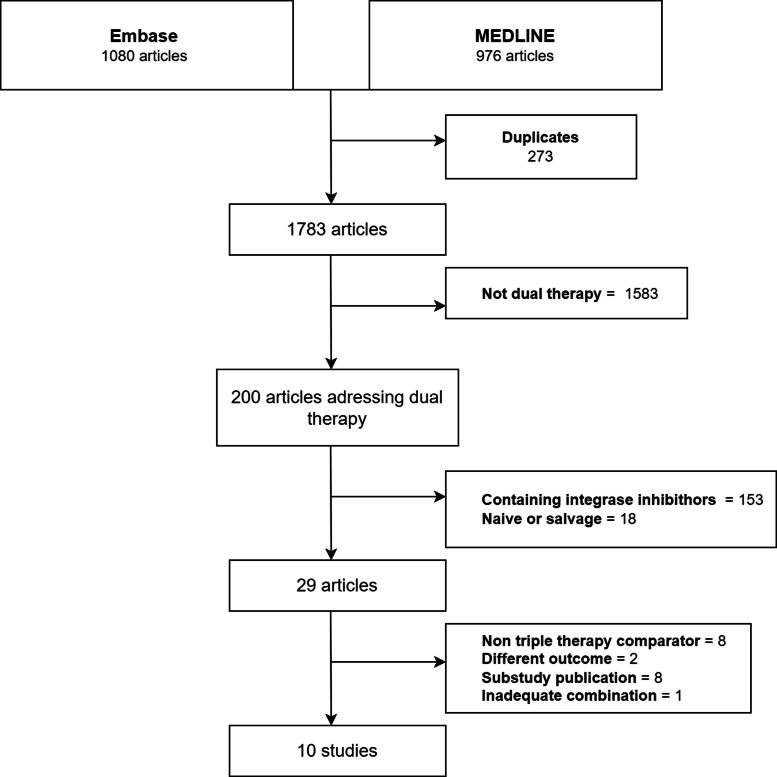
Flowchart of selection of studies.

The exclusion criteria consisted of (a) Non-published studies, (b) Inclusion of naive patients or with non-suppressed HIV viral load in baseline (> 200 copies/mL), and (c) Integrase inhibitors containing dual therapy. Two investigators (MSH and ES) independently assessed the studies for selection criteria, and any discrepancies were jointly evaluated for consensus (complete flowchart in Fig. 1).

### Data extraction and quality assessment

We used a form to extract the data, which consisted of identification (title, author, year), methodologic aspects, clinical and demographic baseline data, drug combination of intervention/exposure and controls, number of participants in each group, and rate of viral failure in and *per-protocol* analysis in 48 weeks. The definition of viral failure was two HIV viral loads above 50 copies/mL measured on separate occasions in most trials.

The Cochrane Risk of Bias 2 tool^27^ was utilized to assess bias in the randomized clinical trials and the Newcastle-Ottawa Scale^28^ for the non-randomized studies. The bias evaluation of the included studies is present in supplementary material.

### Statistical analysis

Given the methodological diversity across the studies included in the analysis, we computed pooled Risk Ratios (RRs) utilizing a random-effects model. RR estimates were presented along with their respective 95 % Confidence Intervals (95 % CIs).

Given virological failure as the outcome of interest, we consider only the indeed exposed population and, therefore, the *per-protocol* analysis data. The outcome was evaluated by amalgamating the absolute frequencies of events using a random-effects approach employing the Mantel-Haenszel method. Study heterogeneity was assessed using χ^2^ statistics and the Higgins I² value. Sensitivity analyses were performed by systematically excluding each study to evaluate the robustness of the data. Statistical analyses were conducted using RevMan Web version 8.1.1.^29^

## Results

The search yielded two hundred studies on dual therapy. After applying the inclusion and exclusion criteria, ten studies remained for the meta-analysis, including nine open label randomized clinical trials and one retrospective cohort study (Fig. 1).

These studies provided data from 1977 patients (865 in simplified therapy and 1112 controls). Table 1 shows a summarized data of studies (including demographics).

**Table 1:** Characteristics of included studies and population.

	Simplification	Control group	Design	N	Male (%)	Age, years	Nadir CD4 (cell/mm³)	CD4 count (cell/mm³)
3TC+PI/r								
ATLAS-M^27^ 2016	3TC+ATV/r	2NRTI+ATV/r	RCT, OL	266	72	44 (36–50)	265 (132–357)	617 (481–781)
DUAL^30^ 2017	3TC+DRV/r	2NRTI+DRV/r	RCT, OL	257	83	43 (36–50)	246 (120–327)	589 (443–762)
Hung et al.^31^ 2019	3TC+LPV/r or 3TC+DRV/r	2NRTI+LPV/r or 2NRTI+DRV/r	Observational	364	91	37 (32–44)	NA	S: 502 (389–693)
								C: 529 (400–675)
OLE^29^ 2015	3TC+LPV/r	2NRTI+LPV/r	RCT, OL	239	69	46 (40–50)	176 (72–266)	610 (440–789)
SALT^28^ 2015	3TC+ATV/r	2NRTI+ATV/r	RCT, OL	286	83	43 (38–51)	212 (108–318)	582 (417–784)
**NNRTI+PI/r**								
Di Cristo et al.^32^ 2020	RPV+DRV/r	Triple therapy	RCT, OL	36	75	S: 44 (41–49)	S: 187 (30–310)	S: 745 (598–1265)
						C: 51 (42–59)	C: 222 (91–350)	C: 670 (549–823)
Negredo et al.^34^ 2009	NVP+LPV/r	2NRTI+PI/r or NNRTI	RCT, OL	66	85	S: 42 (37–47)	NA	S: 471 (385–722)
						C: 42 (38–47)		C: 452 (303–596)
PROBE^33^ 2016	RPV+DRV/r	2NRTI+PI/r	RCT, OL	60	80	S: 49 (10)	S: 233 (163)	S: 615 (271)
						C: 48 (8)	C: 263 (196)	C: 631 (339)
**MVC+PI/r**								
GUSTA^35^ 2017	MVC+DRV/r	Triple therapy	RCT, OL	165	75	49 (41–57)	222 (137–310)	659 (495–923)
MARCH^36^ 2015	MVC+PI/r^a^	2NRTI+PI/r or 2NRTI+MVC	RCT, OL	395	77	43 (10)	213 (162)	617 (251)

^a^Most frequent were ATV/r and LPV/r.

ATV/r, boosted Atazanavir; DRV/r, boosted Darunavir; LPV/r, boosted Lopinavir; MVC, Maraviroc; NVP, Nevirapine; PI/r, boosted Protease Inhibitors; OL, Open-Label; RCT, Randomized Clinical Trial; RPV, Rilpivirine. When pooled data is not available, “S” refers to the “simplification group” and “C” refers to the “control group”. Values in median (IQR) or mean (SD).

There were five studies with 3TC and boosted Protease Inhibitors (bPI), four non-inferiority open-label randomized clinical trials,^30^, ^31^, ^32^, ^33^ and one retrospective cohort.^34^ The bPIs represented in the trials were atazanavir, lopinavir, and darunavir. The simplified group did not show an increased risk of failure with this combination compared to controls (RR = 0.82 [0.43‒1.56], I² = 0 %).

Three small clinical trials comprised the NNRTI and bPI subgroup, two with rilpivirine and boosted DRV^35^^,^^36^ and one with nevirapine and boosted lopinavir.^37^ Virological failures occurred in only one trial^35^ and at a similar rate in both groups.

The bPI subgroup combined with Maraviroc (MVC), composed of two trials,^38^^,^^39^ demonstrated a higher risk of viral failure compared to the control group, with a Relative Risk (RR) of 4.49 ([1.99‒10.11], I² = 0 %).

**Fig. 2:**
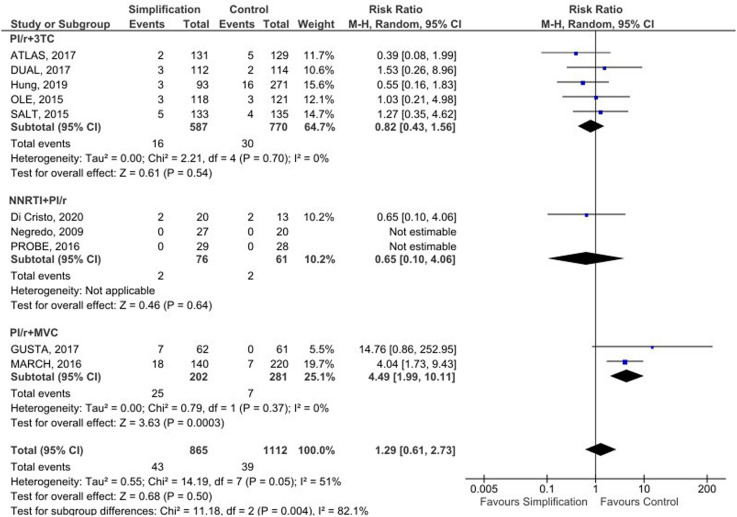
Pooled results.

Overall, there were 43 viral failures in the simplification group and 39 in the control, leading to an RR of failure for simplification versus the control group of 1.29 (0.61‒2.73) with a moderate heterogeneity (I² = 51 %). In the sensitivity analysis, it became clear that the heterogeneity was primarily driven by the MARCH^38^ study; once excluding this trial, the heterogeneity was considered low (I² = 6 %), and the RR for the outcome was 0.91 (0.49‒1.70). Pooled results are available in Fig. 2.

## Discussion

This meta-analysis shows that contemporary HAART simplification in individuals with suppressed viral load is feasible and safe and does not increase the risk of viral failure, even without using INSTI as a component of the therapy. In our analysis, bPI (mainly atazanavir, lopinavir, and DRV) was the core of the simplification regimen. This finding might reflect the pharmacologic limitations of the first generation of PIs as compared to those used in the later years. The frequent co-formulation with ritonavir or cobicistat, which enables once-daily dosing and, consequently, a more patient-friendly regimen could be an example of this improvement.^11^

The most extensively studied antiretroviral combined with bPI in this situation was 3TC. Its use was safe and consistently maintained viral suppression without drug resistance associated mutation development, even when considering M184 V mutation (known to be a hallmark of 3TC resistance) in cases of viral rebound. This drug was the most studied antiretroviral in dual regimens without INSTI and was safe and effective along with the bPI.

Alternatively, other drugs also were combined with bPI. Although based on limited data from small clinical trials, boosted DRV plus rilpivirine and boosted lopinavir plus nevirapine were studied.^35^^,^^37^ These regimens were not inferior to triple therapy and there was no difference regarding viral rebound. This finding suggests their potential effectiveness in clinical practice, although more studies are needed to confirm these findings.

However, the main outcome of interest was different when the CCR-5 co-receptor inhibitor MVC was used with a bPI. This dual therapy resulted in a statistically higher risk of virological failure, as seen in two trials, the MARCH and the GUSTA study.^38^^,^^39^ We can speculate that this finding may reflect a lower adherence associated to a lower genetic barrier in the simplification group as there was no protease mutation detected, and in most patients, HIV remained susceptible to MVC (remained CCR-5 tropic). Additionally, to reinforce this speculation, patients experiencing virological failure in the GUSTA trial exhibited lower serum levels of antiretrovirals. Similar outcomes were also observed in studies evaluating dual therapy regimens with boosted DRV plus raltegravir,^40^^,^^41^ with the later having a bit lower genetic barrier and generally requires twice daily dosing. These findings underscore the importance of formulating dual therapy with drugs that share some pharmacokinetics properties, with fewer tablets and convenient dosing schedules in daily clinical practice, as demonstrated with the other combinations.

Currently, most regimen simplifications include DTG or bPI as the core component. Both regimens have similarities. The most used antiretroviral with them is 3TC.^42^ Additionally, both drugs have proven their effectiveness as for start treatment^43^, ^44^, ^45^ as for maintenance therapy.^30^^,^^31^^,^^46^ Similarly to DTG, bPIs-based regimens are once-daily dosed. Nevertheless, bPI regimens are still more challenging, as they have the potential for more drug-drug interactions^47^ and intolerance related concerns.^48^

Although the results of these trials (with bPI plus 3TC or NNRTI) did not show differences regarding viral failures, a 48-week follow-up period may have some limitations. This length of observation might be insufficient to detect hard outcomes (like death) in the population studied. The limited follow-up duration does not allow for assume the on mortality, which could be linked to the development of comorbidities associated with prolonged exposure to antiretroviral therapy, for instance. On the other hand, reducing treatment toxicity by regimen simplification in well-controlled patients appears to be a logical approach as they continue to age. This meta-analysis suggests that using INSTIs is not essential for this purpose and that could be replaced for a bPI when DTG is not a viable option.

Our study has some weaknesses. First, our analysis focused on individuals indeed exposed to treatment, limiting our findings to the per-protocol population. While this approach aimed to capture those genuinely at risk of virological failure, it inherently excludes an assessment of tolerability, a critical aspect in evaluating treatment outcomes. Nonetheless, due to the nature of reduced drug exposure in this context (taking off one antiretroviral already in use), issues related to drug intolerance are generally not expected in daily clinical practice. Second, the moderate heterogeneity observed in the I² test may reflect differences among the combined drug with bPI (such as 3TC, NNRTIs, and MVC). There was also a lack of standardization in the definition of viral failure in the included studies. The minimum viral load threshold ranged from 50 to 200 copies/mm^3^ on two occasions for defining virological failure, and one trial still included a single viremia measurement above 1000 copies/mL in their definition.^38^ Finally, a significant weakness, albeit limited to one subgroup and with fewer participants, is the small sample size associated with the combination of bPI plus NNRTIs.^35^, ^36^, ^37^

In summary, we have shown that treatment simplification with bPI as a core of the regimen was not only safe but also effective as a maintenance therapy with no difference in viral rebound compared to triple therapy. Therefore, in scenarios such as the increase in dolutegravir resistance or intolerance, simplification regimens utilizing bPIs might be an option. In this way, based on the above trials studied, a combination of bPI as the core of the regimen plus 3TC or NNRTI, albeit less used, will be able to maintain viral load suppression.

## Conflicts of interest

The authors declare no conflicts of interest.

## Appendix Supplementary materials

Image, application 1
