# Supplementary figures and images for: Risk of viral failure after simplification therapy without using integrase inhibitors compared with maintenance of triple antiretroviral therapy: A systematic review and meta-analysis
Source: Braz J Infect Dis. 2024 Nov 17;28(6):104463. doi: 10.1016/j.bjid.2024.104463 (PMC11615594; doi:10.1016/j.bjid.2024.104463)

**SUPPLEMENTARY MATERIAL**

Figure 3.

Quality Assessment


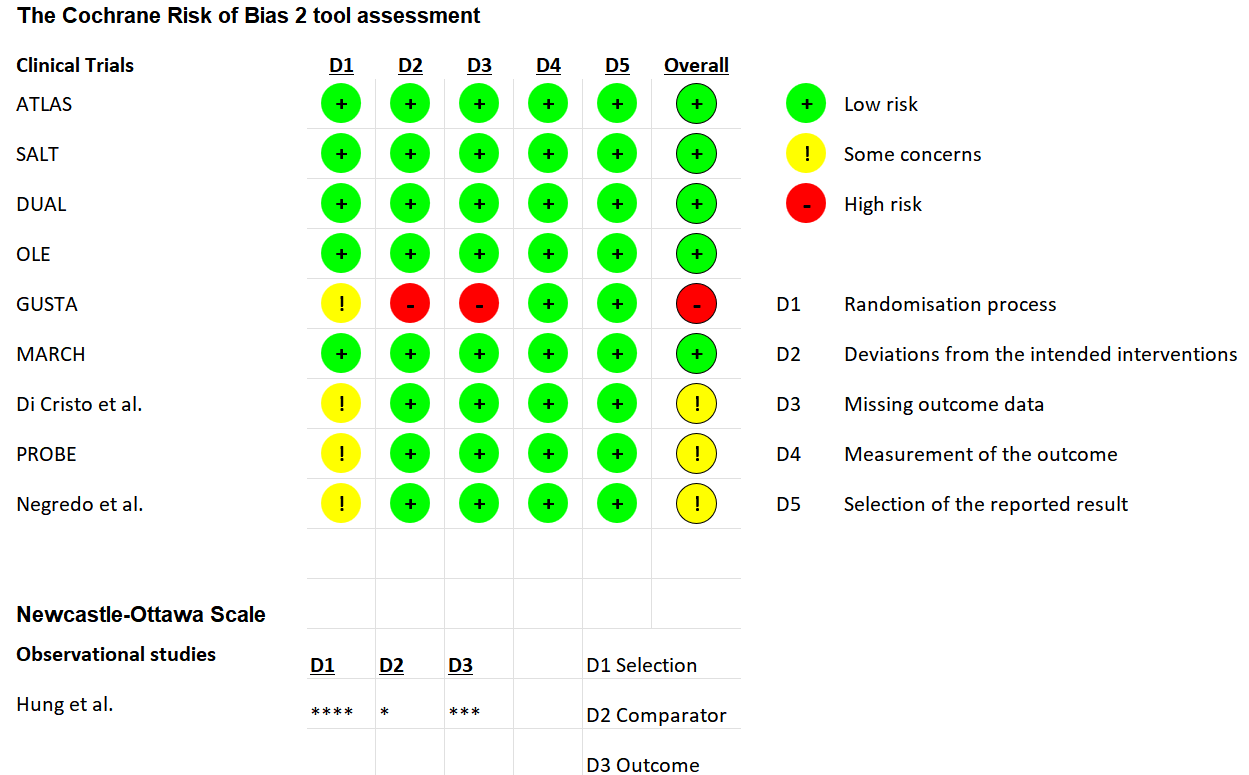

Supplement: Supplementary file 1 [file mmc1.docx]
